# Economic evaluation of hearing aid use and quality of life in older adults with hearing impairment in India

**DOI:** 10.3389/fmedt.2026.1800134

**Published:** 2026-05-12

**Authors:** Rakesh Kumar Sahoo, Krushna Chandra Sahoo, Urmi Pattanayak, Abhinav Sinha, Abhisek Jena, Lanu Wanboy Aimol, Kavitha Rajsekar, Debdutta Bhattacharya, Sanghamitra Pati

**Affiliations:** 1 Health Technology Assessment Regional Resource Hub (HTAIn), ICMR-Regional Medical Research Centre, Bhubaneswar, Odisha, India; 2 Ali Yavar Jung National Institute of Speech and Hearing Disabilities (Divyangjan), Regional Center, Janla, Bhubaneswar, India; 3 Health Technology Assessment in India (HTAIn), Department of Health Research, Ministry of Health & Family Welfare, New Delhi, Govt. of India

**Keywords:** cost–utility analysis, EQ-5D-5L, hearing aids, hearing impairment, quality-adjusted life years (QALYs)

## Abstract

Hearing impairment is a growing public health challenge among older adults in India, with low uptake of hearing aids despite potential benefits for communication, social participation, and health-related quality of life (HRQoL). This study evaluated the cost–utility of hearing aid provision for older adults (≥60 years) with hearing impairment in India to inform policy and financing decisions. A cost–utility analysis compared hearing aid use with no hearing aid over a 3-year decision tree and a 15-year Markov model. Data were collected in 2023 from 636 participants (276 users, 360 non-users). HRQoL was measured using EQ-5D-5L and converted to QALYs with the India value set. Costs were based on the government ceiling price for digital behind-the-ear hearing aids (₹8,000 per ear), modelled for unilateral and bilateral use. ICERs and budget impact were estimated. Mean EQ-5D-5L utility was higher among hearing aid users than non-users (0.832 vs. 0.601), with corresponding QALYs of 5.824 vs. 4.207 (incremental gain 1.617 QALYs). In the 3-year decision tree, ICERs were ₹5,419/QALY (one-year) and ₹10,359/QALY (two-year). In the discounted 15-year Markov model, ICERs were ₹3,076/QALY (one-year) and ₹5,971/QALY (two-year). Budget impact was ₹1,230 crore over 3 years (₹410 crore annually) for the modelled scale-up among older adults. Hearing-aid provision for older adults with hearing impairment in India is highly cost-effective in short- and long-term modelling. Prioritising access within healthy ageing and universal health coverage, alongside strengthened assessment, fitting, counselling, and follow-up, could maximise real-world benefits.

## 1 Introduction

Hearing impairment is a major and growing public health concern among older adults. Globally, hearing loss is a leading contributor to years lived with disability, and the burden rises steeply with age (1). Age-related hearing loss is highly prevalent in later life and ranks as the fifth leading cause of disability worldwide, imposing substantial economic and societal costs. Progressive hearing loss impairs speech perception and communication essential for social participation and independence, and is associated with reduced quality of life and increased risk of depression (2, 3). The burden of hearing impairment is projected to rise in low- and middle-income countries, including India, driven by population ageing and increasing chronic disease prevalence. By 2,050, adults aged ≥60 years are expected to comprise nearly one-fifth of India's population (4). Within this group, hearing difficulty is common and often co-occurs with health and social vulnerabilities. The World Health Organization (WHO) estimates that approximately 63 million people in India have significant hearing impairment, with prevalence reaching about 67% among older adults (5, 6). These trends underscore the importance of prioritizing hearing health within healthy ageing policies and programs (7). Moreover, untreated hearing loss is a “silent epidemic” with significant clinical and social consequences. It is consistently associated with accelerated cognitive decline and dementia, with Indian evidence indicating a 1.69-fold higher risk of cognitive impairment among affected older adults (6). Communication difficulties associated with hearing impairment contribute to social isolation, reduced participation, and poorer health-related quality of life (HRQoL), and have also been identified as a potentially modifiable risk factor for dementia, with prospective studies demonstrating an association between hearing loss and incident dementia (8, 9). The EQ-5D-5L has been increasingly used in India to assess HRQoL among people with hearing impairment, providing a concise, standardised measure that captures mobility, self-care, usual activities, pain/discomfort, and anxiety/depression to inform accessibility and rehabilitation planning. Collectively, these findings underscore the wide-ranging impact of hearing loss on well-being, functional independence, and long-term health outcomes.

Hearing aids remain the primary rehabilitative intervention for age-related hearing impairment. Hearing aid use improves hearing-related functioning and HRQoL by mitigating the social and psychological consequences of sensorineural hearing loss (10). However, despite demonstrated benefits, hearing aid uptake and sustained use remain low. Recent evidence indicates that although more than 400 million people globally could benefit from hearing aid use, only about 17% currently use these devices; in India, only 5% of older adults with hearing difficulty report hearing aid use, and evidence syntheses suggest that up to 40% of adults fitted with hearing aids either do not use them or do not achieve optimal long-term benefit (5–10). Adoption is influenced by a complex interplay of audiological factors (such as severity of hearing loss) and non-audiological factors, including perceived need, stigma, device comfort, service accessibility, affordability, socioeconomic status, and health system constraints (11, 12). In India, population-based analyses indicate very low hearing aid use among older adults who report hearing difficulty, pointing to a substantial unmet need (7). Limited availability of hearing health services in some regions, uneven service delivery capacity, and high out-of-pocket costs borne by households further hinder timely adoption and continuity of care (13). Given competing healthcare priorities and constrained public resources, decisions to expand hearing aid provision and related services increasingly require robust evidence of economic evaluation to justify value for money.

Cost–utility analysis (CUA) compares costs and outcomes, such as quality-adjusted life years, of alternative interventions. This CUA was chosen because hearing aids affect communication, daily functioning, social participation, well-being, and quality of life. A CUA also permits comparison with other health interventions within the Indian HTA context. In this study, utilities were derived using the Indian EQ-5D-5L value set, developed from population preferences in India, thereby improving the local validity and policy relevance of QALY estimation (14). However, evidence from LMICs remains limited, and uncertainty persists regarding locally valid utility estimates and real-world implementation costs for hearing healthcare (15). Against this backdrop, the present study conducted an India-specific cost–utility evaluation of hearing aid devices for older adults with hearing impairment, using primary EQ-5D-5L data. The analysis assessed whether hearing aid provision improves HRQoL sufficiently to represent excellent value for money compared with non-use, hypothesising that use would be associated with higher utility, greater QALY gains, and a favourable incremental cost-effectiveness ratio (ICER) in an Indian setting.

## 2 Methods

### 2.1 Population, intervention, comparators, outcomes, and time-horizon (PICOT)

An economic evaluation of hearing aid provision for older adults with hearing impairment in India was conducted using cost–utility analysis. The study included older adults with hearing impairment in India; participants were recruited from eligible clinic attendees and community sources using predefined inclusion criteria, with explicit exclusion criteria applied to those without confirmed hearing loss or lacking consent. For local demographics, gender representation was reported or balanced. The population included presbycusis and other hearing loss aetiologies (including congenital cases if present), and data on comorbidities and other healthcare expenditures were collected to assess competing priorities (chronic diseases) that could affect hearing aid uptake and quality of life.

Using the PICOT framework: P: adults aged ≥60 years with hearing impairment, classified as mild (20–34 dB), moderate (35–64 dB), or severe (≥65 dB); I: hearing aid use, modelled separately for unilateral (monaural) and bilateral (binaural) fittings; C: no hearing aid (non-users); O: incremental cost per quality-adjusted life year (QALY) gained, expressed as the incremental cost-effectiveness ratio (ICER); T: a base-case 3-year decision tree model, with a scenario analysis extrapolating outcomes over 15 years using a cohort Markov model with annual cycles. The evaluation was reported in accordance with the Consolidated Health Economic Evaluation Reporting Standards—CHEERS 2022 (16).

### 2.2 Data collection and outcome measurement

The study was conducted in Bhubaneswar, Odisha, India, with a model-based cost-effectiveness approach supplemented by primary survey data. Participants were drawn from a structured questionnaire-based survey of hearing-impaired older persons (*n* = 636), including both hearing aid users (*n* = 276) and non-users (*n* = 360), conducted in partnership with the Ali Yavar Jung National Institute of Speech and Hearing Disabilities. Individuals diagnosed with hearing impairment above the age of 60 were eligible for inclusion. Hearing impairment severity categories were defined using WHO hearing threshold cut-offs: mild (20–34 dB), moderate (35–64 dB), and severe (≥65 dB). These categories were based on the WHO hearing-loss grading using the better-ear hearing threshold. For model parsimony, the WHO moderate and moderately severe grades were combined into a single 35–64 dB category (17).

The HRQoL was measured using EQ-5D-5L, and utilities were created with the India EQ-5D-5L value set. The structured questionnaire contained a module on resource use and expenditures, as well as the EQ-5D-5L instrument. The EQ-5D-5L is a standardised generic preference-based measure that includes five dimensions: mobility, self-care, typical activities, pain/discomfort, and anxiety/depression, each with five response levels. Responses were translated to utility values using the Indian EQ-5D-5L value set (18), which was created for use in health technology assessment and resource allocation in India.

### 2.3 Costing approach

The economic evaluation included direct medical, direct non-medical, and device-related capital costs from a societal perspective, regardless of payer. A three-year time horizon was used to reflect hearing aid lifespan and initial and recurring costs like maintenance and follow-up. Costing used primary and secondary data for practicality. The digital behind-the-ear (BTE) hearing aid costs INR 8,000 per ear, including taxes and a three-year warranty, based on government ceiling rates from the Government e-Marketplace (GeM) and the Delhi Government Health Scheme (19). For continued users, replacement and re-evaluation were assumed at the end of each three-year model cycle. From a 2023 structured primary survey of 636 participants, consultation, diagnostics, transportation, and maintenance costs were calculated.

### 2.4 Model structures

The decision tree compared hearing aid use vs. no hearing aid use, with the intervention arm disaggregated into unilateral and bilateral fitting pathways. Expected costs and QALYs for each pathway were estimated using observed utilities and cost inputs, and incremental results were calculated as differences between strategies. A cohort Markov state-transition model was developed with three mutually exclusive health states: mild, moderate, and severe hearing impairment over a 15-year horizon with annual cycles. Transitions allowed progression from mild to moderate or severe, and from moderate to severe, with state occupancy determined by a transition probability matrix. Cohort-specific probabilities were specified separately for hearing aid users (UHA) and non-users (NUHA), including probabilities of remaining in a given state or progressing to a worse state. The model assumed a 3-year hearing aid lifespan, with device costs repeated every three years for continuous users and additional re-evaluation costs applied if hearing status changed. Outputs were generated as discounted estimates and reported from a health system perspective. For clinical interpretability, these Markov states represent hearing-loss severity status over time rather than treatment states. Thus, at each annual cycle, participants could remain in the same severity category or progress to a worse severity category according to the transition probability matrix. The 3-year hearing aid lifespan assumption was operationalized as a replacement interval for continued users and was aligned with the government reimbursement specification and warranty period for Digital BTE devices, rather than implying uniform device failure at exactly 3 years.

### 2.5 QALY estimation and incremental analysis

Utilities derived from EQ-5D-5L were converted into QALYs by applying utility weights to time. The report's approach also links QALY projection to remaining life expectancy assumptions (e.g., using life expectancy for estimating years over which utility is accrued). Incremental cost-effectiveness was expressed as an ICER (INR per QALY gained), calculated as the difference in total costs divided by the difference in total QALYs between hearing aid and no-hearing-aid strategies.

### 2.6 Discounting, WTP threshold, and validation

In economic evaluation, time preference reflects the assumption that costs and health gains occurring in the future are valued less than those occurring in the present; accordingly, future costs and QALYs were discounted to present values. In line with the Indian HTA Reference Case, a 3% annual discount rate was applied to both costs and outcomes. To interpret whether the estimated incremental cost-effectiveness ratio (ICER) represented good value for money, a willingness-to-pay (WTP) threshold was applied. In the present study, the ICER was compared against a contextual benchmark of 1× India's per-capita GDP per QALY gained. This benchmark was used as a pragmatic interpretive threshold, as India does not yet have a formally adopted national cost-effectiveness threshold. The findings were therefore interpreted in relation to this benchmark to assess whether hearing aid provision could be considered cost-effective in the Indian setting. Model validation was undertaken by comparing model structure and outputs with observed primary survey estimates, administrative price sources, and alternative modelling assumptions relevant to the Indian setting.

### 2.7 Budget impact analysis

A national budget impact estimate was generated using Census-based projections for the ≥60 population and prevalence inputs from LASI, applying an estimated proportion requiring hearing aids and distributing expected demand across severity/fitting categories as specified. The budget impact analysis used projected population counts for adults aged ≥60 years, prevalence estimates for hearing impairment, and the assumed distribution of one-ear vs. two-ear fitting need, as reported in the outcome report.

## 3 Results

### 3.1 Study sample and hearing loss severity

The analysis included 636 older adults with hearing impairment, comprising 276 hearing aid users and 360 non-users. Among hearing aid users, hearing loss was classified as mild in 36, moderate in 122, and severe in 118 participants. Among non-users, hearing loss was classified as mild in 153, moderate in 131, and severe in 76 participants (Supplementary Table S1).

### 3.2 Direct medical costs and hearing aid fitting costs (INR per person; 3 years)

Direct medical costs comprised hearing-related consultation costs and diagnostic investigation/testing costs. Consultation referred to clinical visits related to hearing assessment and hearing-aid care, whereas investigation referred to diagnostic hearing evaluation/testing. These costs were derived from the primary structured questionnaire survey and aggregated over the 3-year study horizon. Consultation costs were applied once over 3 years, while diagnostic investigation/testing costs were applied twice in the first year and once in each subsequent year.

Over the 3 years, the average direct medical cost was INR 4,368 per hearing-aid user and INR 1,583 per non-user, with diagnostic investigation/testing contributing the largest proportion in both groups. Applying the government ceiling price of INR 8,000 per ear for a digital behind-the-ear device (3-year warranty), the estimated total 3-year per-person cost was INR 12,368 for unilateral fitting and INR 20,368 for bilateral fitting, inclusive of direct medical costs (Table 1).

**Table 1:** Direct medical costs and hearing aid fitting costs over three years (INR per person).

Cost item	Hearing aid users	Non-users	Unilateral (single ear)	Bilateral (both ears)
Consultation	111	131	—	—
Investigations	4,257	1,452	—	—
Total direct medical cost	4,368	1,583	4,368	4,368
Device cost	—	—	8,000	16,000
Total fitting cost (device + direct medical)	—	—	12,368	20,368

Primary survey, 2023; Government ceiling rate for device cost.

### 3.3 Health-related quality of life and QALYs

EQ-5D-5L utility scores were higher among hearing aid users than non-users across all severity categories. Overall, mean utility was 0.832 among users compared with 0.601 among non-users. The corresponding average QALYs were 5.824 and 4.207, respectively (Table 2).

**Table 2:** EQ-5D-5L utility scores and QALYs by hearing loss severity.

Severity category	Users: Utility	Users: QALYs	Non-users: Utility	Non-users: QALYs
Severe	0.904	6.328	0.614	4.088
Moderate	0.807	5.649	0.598	4.431
Mild	0.763	5.460	0.592	4.081
Overall	0.832	5.824	0.601	4.207

Primary survey, 2023; utilities derived from EQ-5D-5L (India value set).

### 3.4 Cost-utility results (decision tree+markov) and budget impact analysis

**Figure 1:**
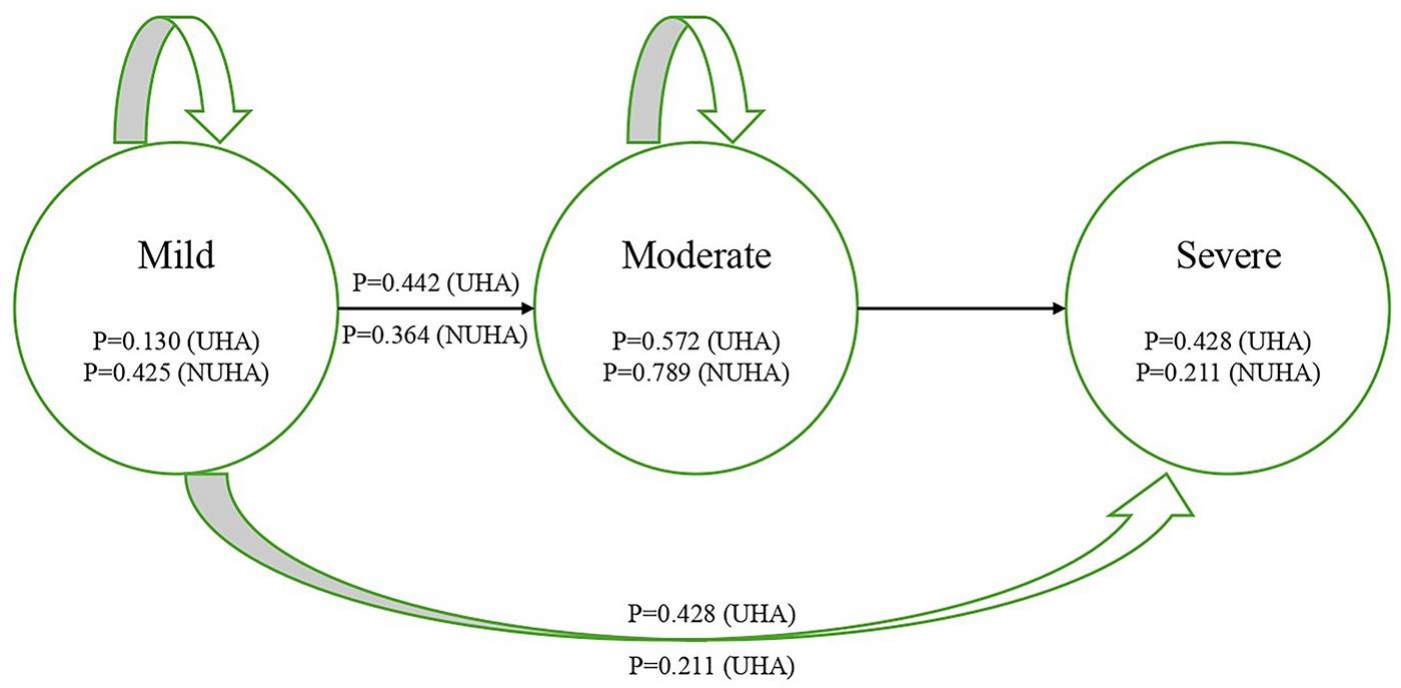
Markov model structure and transition probabilities (15-year horizon).

In the base-case (3-year decision tree) analysis, hearing aid provision increased QALYs at higher costs compared with no hearing aid, yielding ICERs of INR 5,419 per QALY for unilateral fitting and INR 10,359 per QALY for bilateral fitting. For longer-term extrapolation, a three-state cohort Markov model (mild, moderate, severe) was applied over 15 years with annual cycles; the model structure and transition probabilities are presented in Figure 1, and the discounted health-system perspective ICERs were INR 3,076 per QALY (unilateral) and INR 5,971 per QALY (bilateral) fitting. The budget impact analysis estimated the national financial implications of scaling up hearing aid provision, projecting a total 3-year budget requirement of INR 1,230 crore, equivalent to approximately INR 410 crore annually (Table 3).

**Table 3:** Cost-utility (decision tree + markov) and budget impact analysis results.

Domain	Measure	Hearing aid (Unilateral)	Hearing aid (Bilateral)	Comparator/Overall
Base-case (3-year decision tree)	Total cost (INR)	11,420	19,420	No hearing aid: 2,669
	QALYs	5.824	5.824	No hearing aid: 4.207
	Incremental cost (INR)	8,751	16,751	—
	Incremental QALYs	1.617	1.617	—
	ICER (INR/QALY)	5,419	10,359	—
Scenario (15-year Markov; discounted)	Use hearing aid: Cost (INR)	7,676,173	12,641,356	—
	Use hearing aid: QALYs	2,613	2,613	—
	No hearing aid: Cost (INR)	2,401,354	2,401,354	—
	No hearing aid: QALYs	898	898	—
	Incremental cost/QALYs	5,274,819/1,715	10,240,002/1,715	—
	ICER (INR/QALY)	3,076	5,971	—
Budget impact (3 years)	Older adults aged 60+ (projected)	—	—	14.9 crore
	Prevalence of hearing impairment	—	—	10% (1.49 crore)
	Estimated need for hearing aids	—	—	4.7% (70,030)
	Need for fitting	35% (24,510)	65% (45,520)	—
	Cost per person (3 years)	12,368	20,368	—
	Total cost (3 years)	303 crores	927 crores	All fittings: 1,230 crores
	Annual cost	—	—	410 crores

INR, Indian Rupees; QALY, quality-adjusted life year; ICER, incremental cost-effectiveness ratio; unilateral, hearing aid fitting for one ear; bilateral, hearing aid fitting for both ears; comparator, no hearing aid use; base-case, primary analysis using the 3-year decision-tree model; scenario, additional long-term analysis using the 15-year cohort Markov model; discounted, future costs and QALYs adjusted to present values using the applied discount rate; crore = 10 million (10,000,000).

## 4 Discussion

This cost-utility analysis in India demonstrates that providing hearing aids to older adults with hearing impairment significantly enhances quality-adjusted life years (QALYs), yielding an incremental gain of 1.617 QALYs. The base-case analysis, utilizing a 3-year decision tree from a societal perspective, reveals that hearing aid users experience higher utility (0.832) compared to non-users (0.601). The costs associated with unilateral and bilateral fittings are ₹8,751 and ₹16,751, respectively, resulting in low incremental cost-effectiveness ratios (ICERs) of ₹5,419/QALY and ₹10,359/QALY. In a 15-year Markov scenario, ICERs further decrease to ₹3,076/QALY for unilateral and ₹5,971/QALY for bilateral fittings, indicating that long-term benefits outweigh costs. Overall, the findings suggest that hearing aid provision is a cost-effective investment in India, aligning with various cost-effectiveness benchmarks and supporting the value of hearing aids for enhancing the well-being of older adults.

Our results align with international evidence that hearing aids improve health-related quality of life and can be cost-effective, particularly when costs are contained through public procurement and when benefits persist over multiple years. Global analyses commissioned to support WHO ear and hearing care planning have similarly concluded that scaling access to hearing technologies and related services can yield substantial health gains and favourable economic returns when implemented at scale (20). In the Indian context, however, the reference to public procurement should be interpreted within a mixed financing landscape. Hearing-aid access is not financed through a single universal pathway: eligible beneficiaries may receive assistive devices through government or social welfare channels, whereas broader Indian assistive-technology evidence suggests that many users still obtain assistive products at their own expense and that inability to afford them remains a common barrier to access (21–23). The very low ICERs observed here are partly driven by two contextual factors relevant to India: the use of a publicly benchmarked ceiling price for devices, and a large utility differential between users and non-users measured directly in an older adult population using the Indian EQ-5D-5L value set (24).

The magnitude of the observed utility gap warrants careful interpretation. Generic preference-based instruments such as EQ-5D may under-capture certain sensory and participation benefits compared with hearing-specific measures; however, empirical work also suggests that generic measures can show improvements when hearing loss meaningfully affects daily functioning and psychosocial domains (5). In this study, higher utility among hearing aid users could reflect the direct effect of amplification on communication, social engagement, and emotional well-being, but it may also reflect residual confounding (e.g., socioeconomic status, comorbidity profiles, care-seeking patterns) inherent to cross-sectional comparisons of users vs. non-users. This is especially relevant because observed direct medical costs were higher among users than non-users over three years (₹4,368 vs. ₹1,583), potentially indicating differences in health service utilization and diagnostic evaluation intensity that may correlate with need, perceived health status, and access to care, all of which are established determinants of health service utilization (25, 26). Future analyses that adjust for baseline differences (e.g., propensity score methods or regression-based net benefit frameworks) would strengthen causal interpretation of the QALY gain and improve transferability across settings.

Beyond HRQoL, hearing loss is increasingly recognised as a major contributor to later-life disability and broader adverse outcomes. WHO has highlighted a large unmet need and low coverage of hearing care globally, emphasising amplification as a core rehabilitative intervention within integrated ear and hearing care (27, 28). The broader relevance of hearing interventions is also underscored by dementia prevention frameworks in which hearing loss is treated as a modifiable risk factor, and randomised evidence suggests that structured hearing interventions may reduce cognitive decline in specific higher-risk subgroups (29). While cognitive outcomes were not modelled here, these pathways strengthen the policy rationale for improving hearing aid access and continuity of use, especially given India's rapid population ageing (30).

From a policy perspective, the combination of low ICERs and a quantified budget impact creates a clear decision-making package. The budget impact analysis estimates that scaling hearing aid provision to the modelled level of need would require approximately INR 1,230 crore over three years (about ₹410 crore annually), with the majority of the cost attributable to bilateral fittings, given the assumed demand distribution. These figures provide a concrete financing benchmark that can be used to assess affordability, to plan phased expansion, and to negotiate procurement and service delivery models (e.g., bundled pricing for device plus fitting plus follow-up). Importantly, budget impact should be interpreted as the resource envelope required not only for devices but also for the service platform needed to ensure sustained use (audiology workforce, fitting, counselling, batteries/maintenance pathways, and follow-up), some of which may not be fully captured in participant-reported expenditure. The findings are directly relevant to the current program architecture. Under the National Programme for Prevention and Control of Deafness (NPPCD), individuals requiring intervention are referred for specialist/audiology services, while hearing aids themselves are commonly linked to social welfare provision for eligible beneficiaries rather than being financed as a universal entitlement within the health programme; policy documents have also noted the absence of a dedicated budget line for hearing aids (29, 30). This mixed arrangement helps explain why both public procurement and household affordability matter in practice. In this context, public procurement at ceiling rates can materially improve value for money, but implementation success will depend on reducing non-price barriers, such as stigma, perceived benefit, device comfort, and continuity of service, that drive low uptake and discontinuation. In other words, the “cost-effective” result is conditional on real-world adherence and sustained use; program design should therefore include counselling, troubleshooting, and accessible follow-up to protect the expected health gains.

The study's strength lies in its use of primary Indian utility data with the Indian EQ-5D-5L tariff, enhancing local relevance and avoiding foreign preference weights. It effectively combines a decision tree with Markov extrapolation to model hearing impairment progression and device replacement. Additionally, it presents both cost-effectiveness and budget impact, aligning with HTA best practices. However, limitations include reliance on self-reported data and standard modelling assumptions, which may introduce uncertainty. Future research should focus on longitudinal follow-up, detailed resource measurement, and comparative evaluations to enhance policy relevance and address equity impacts. Overall, it demonstrates strong value for hearing aid provision in India.

## 5 Conclusion

Hearing aid provision for older adults with hearing impairment represents a highly cost-effective intervention in the Indian context. The evaluation indicates consistent improvements in health-related quality of life, with favourable results sustained across short- and longer-term modelling and under alternative assumptions. These findings support prioritising hearing aid access within healthy ageing and universal health coverage strategies. Population-level impact will be maximised when device provision is paired with a strengthened service platform for assessment, fitting, counselling, follow-up, and support for continued use.

## Data Availability

The raw data supporting the conclusions of this article will be made available by the authors, without undue reservation.
