# Supplementary material for: Economic evaluation of hearing aid use and quality of life in older adults with hearing impairment in India
Source: Front Med Technol. 2026 May 12;8:1800134. doi: 10.3389/fmedt.2026.1800134 (PMC13201482; doi:10.3389/fmedt.2026.1800134)
Supplement: Supplementary file 1 [file Table1.docx]

Supplementary Table1: Study sample by hearing aid use and hearing loss severity

| **Group** | **Total (n)** | **Mild (n)** | **Moderate (n)** | **Severe (n)** |
| --- | --- | --- | --- | --- |
| Hearing aid users | 276 | 36 | 122 | 118 |
| Non-users | 360 | 153 | 131 | 76 |
| Total | 636 | 189 | 253 | 194 |

Source: Primary survey, 2023
